# Molecular Insights into ABA-Mediated Regulation of Stress Tolerance and Development in Plants

**DOI:** 10.3390/ijms26167872

**Published:** 2025-08-15

**Authors:** Naeem Khan

**Affiliations:** Institute of Food and Agricultural Sciences, Department of Agronomy, University of Florida, Gainesville, FL 32611, USA; naeemkhan@ufl.edu or naeemkhan001@gmail.com

**Keywords:** abscisic acid, CRISPR/Cas9, omics technologies, stress adaptation, gene expression regulation, crop resilience

## Abstract

Abscisic acid (ABA) is a central phytohormone that orchestrates plant responses to abiotic stresses, such as drought, salinity, and extreme temperatures, while also influencing growth and development. The regulatory networks underpinning ABA-mediated stress tolerance have been the focus of intensive research, revealing sophisticated mechanisms of biosynthesis, signal transduction, and gene regulation. Recent advances in genetic, genomic, and biochemical approaches have illuminated the complexity of ABA’s interactions with other hormonal and environmental signaling pathways, providing a multidimensional understanding of plant adaptation. This review critically synthesizes current knowledge on ABA’s regulatory frameworks, identifies key gaps in our understanding, and discusses the potential integration of omics and emerging technologies to uncover new insights. By offering a comprehensive synthesis of recent findings, this paper aims to stimulate further research into the interplay of ABA with other signaling pathways, highlighting its translational potential for crop improvement under changing environmental conditions.

## 1. Introduction

Plants, as sessile organisms, are frequently subjected to a wide range of abiotic stresses, such as drought, salinity, and temperature fluctuations, which collectively threaten agricultural productivity and global food security. To survive under these adverse conditions, plants have developed intricate physiological and molecular mechanisms to detect, signal, and respond to environmental cues [1,2,3]. These mechanisms are mediated by a network of signaling molecules, among which ABA plays a pivotal role. ABA is not only crucial for immediate stress responses, such as stomatal closure, but also for long-term adaptive processes like seed dormancy and gene expression reprogramming. ABA’s function as a central regulator of stress tolerance is rooted in its dual ability to mediate rapid physiological responses and orchestrate transcriptional networks [4,5]. Under conditions of drought or high salinity, for instance, ABA accumulates in guard cells, triggering ion channel activities that result in stomatal closure to minimize water loss. Simultaneously, ABA modulates gene expression in various tissues, enhancing the production of stress-protective proteins and metabolites [6,7]. Despite decades of research, the full complexity of ABA’s regulatory network remains a topic of intense investigation, particularly its interactions with other signaling molecules and environmental factors.

This review aims to synthesize the current understanding of ABA-mediated stress tolerance, providing an in-depth analysis of recent developments and future directions in the field. In recent years, there has been a surge in high-resolution molecular studies and omics-based approaches that have transformed our understanding of ABA signaling networks. However, an integrated review that connects these findings to practical strategies for enhancing crop resilience remains lacking. The review focuses on the mechanisms of ABA biosynthesis, signal perception, and transcriptional regulation, critically evaluating recent findings and their implications for agricultural practices. It will also examine the interactions between ABA and other signaling pathways, offering insights into the complex regulatory networks that control plant adaptation to various stressors. Unlike previous reviews, this work emphasizes a systems-level perspective that bridges molecular mechanisms with translational applications, particularly in the context of climate-resilient agriculture. In doing so, this review will identify key research gaps, such as the integration of ABA signaling with biotic stress responses and the optimization of ABA manipulation strategies, and propose potential future directions to overcome these challenges. By advancing our understanding of ABA’s multifaceted role in plant stress biology, this paper seeks to inform new strategies for improving crop resilience, ultimately benefiting agricultural production and food security in the face of climate change. ABA, as a master regulator of stress tolerance, holds immense potential for developing climate-resilient crops. However, harnessing this potential requires a holistic understanding of its regulatory networks and a systems-level approach to plant stress biology.

### 1.1. Emerging Themes in ABA Research

Recent investigations into ABA biology have unveiled several critical and evolving themes that are reshaping our understanding of its role in plant stress responses. These emerging areas of research are driven by advances in molecular tools, genomics, and a deeper integration of ABA’s role in plant development, stress tolerance, and interactions with other signaling networks.

One of the central themes is the biosynthesis and catabolism of ABA. Through innovative biochemical and molecular techniques, researchers have gained a deeper understanding of the key enzymes involved in ABA metabolism, particularly 9-cis-epoxycarotenoid dioxygenase (NCED), which plays a pivotal role in ABA biosynthesis, and CYP707A, which is critical for ABA catabolism [8,9]. Despite these advances, fundamental questions persist about the regulation of these pathways under fluctuating environmental conditions. In particular, how plants fine-tune the balance between ABA synthesis and degradation in response to stress is still not fully understood. The integration of environmental signals, such as water availability and light, with the regulation of NCED and CYP707A remains a topic of intense investigation [10,11]. Moreover, environmental cues may not only influence the activity of these enzymes but also regulate the stability and localization of ABA precursors and metabolites.

Another key theme revolves around signal transduction pathways. The identification of PYR/PYL/RCAR ABA receptors has been a groundbreaking development in ABA research. These receptors, along with their downstream interactions with protein phosphatases 2Cs (PP2Cs) and SNF1-related protein kinase 2s (SnRK2s), have revolutionized our understanding of ABA signal transduction [12,13]. This signaling cascade activates a variety of stress-responsive genes, which are essential for plant adaptation [14,15]. However, the regulatory networks downstream of ABA receptors, especially in relation to stress-specific gene activation, remain incompletely mapped. How these signaling components are differentially regulated in various stress conditions, and how they integrate with other signaling networks, requires further exploration. Researchers are particularly interested in how ABA influences the transcriptomic reprogramming that occurs during drought, salinity, and temperature stress, and what regulatory layers, such as chromatin modifications or RNA stability, contribute to this process.

**Figure 1:**
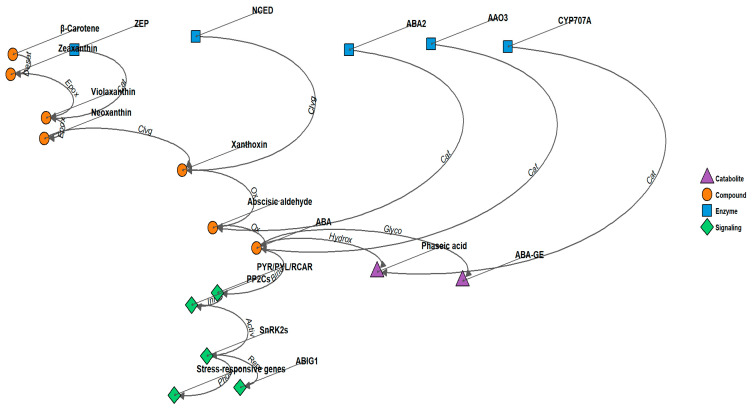
ABA signaling pathway and ABIG1 function in Arabidopsis. The model illustrates the role of ABIG1 in mediating ABA-induced growth inhibition and leaf senescence. Drought stress triggers ABA biosynthesis, leading to increased ABIG1 transcription. ABIG1 acts downstream of the core ABA signaling components (PYR/PYL/RCAR receptors, PP2Cs, and SnRK2s) to specifically regulate shoot growth restriction and promote leaf senescence, without affecting other ABA responses such as seed germination or root growth inhibition. Arrows indicate positive regulation, while bars represent negative regulation. Desat: Desaturation, Epox: Epoxidation, Cat: Catalysis, Clvg: Cleavage, Ox: Oxidation, Hydrox: Hydroxylation, Glyco: Glycosylation, Bind: Binding, Inhib: Inhibition, Activ: Activation, Phos: Phosphorylation, Repr: Transcriptional Repression.

Crosstalk between ABA and other signaling pathways is another prominent and highly dynamic area of research. ABA does not act in isolation but interacts with various other plant hormones such as auxins, gibberellins, ethylene, and cytokinins. These interactions are critical for coordinating plant responses to complex environmental challenges [16,17]. The molecular basis for this crosstalk, particularly how ABA integrates with other phytohormones in a stress-specific context, is an area ripe for further exploration. For instance, ABA often functions antagonistically with gibberellins, inhibiting seed germination, while it works synergistically with ethylene in the regulation of stomatal closure [18,19]. Understanding how these interactions are balanced to ensure optimal plant performance under varying stress conditions will be a key challenge for the next decade of ABA research (Figure 1).

A more recent discovery has been ABA’s involvement in modulating biotic stress responses [20,21]. Traditionally, ABA was primarily associated with abiotic stress responses; however, recent findings have revealed that ABA also plays a role in plant immunity [22]. This dual role in both biotic and abiotic stress responses offers exciting new possibilities for crop protection and resilience. As pathogens, herbivores, and abiotic stressors often occur simultaneously in nature, understanding how ABA balances these dual roles could lead to the development of crops with enhanced tolerance to both environmental stresses and diseases [23,24].

### 1.2. Challenges in ABA Signaling Research

Despite the many advances in ABA research, several critical gaps remain. One of the most pressing questions is how plants prioritize and integrate ABA signaling with other hormonal and environmental pathways, particularly under simultaneous stresses. Plants face multiple, often conflicting, environmental signals, and deciphering how ABA signaling interacts with other stress response networks, like those involving jasmonic acid or salicylic acid, is a complex but essential task. Additionally, research into the structural and functional diversity of ABA receptors across different plant species is still in its infancy [25,26]. While studies in model organisms such as *Arabidopsis* have revealed much about ABA receptor function, it remains unclear how these receptors may differ in agriculturally important species, particularly those with more complex genomes and variable stress responses. These receptor differences could provide insight into species-specific responses to ABA and stress tolerance.

Another challenge is the potential for manipulating ABA signaling to enhance stress tolerance without negatively affecting growth or yield. While ABA has proven crucial in stress responses, its manipulation in crops has often resulted in undesirable effects, such as inhibited growth or delayed development [27]. As such, it is essential to develop more sophisticated strategies that can modulate ABA signaling specifically in response to stress conditions, while maintaining normal plant growth and productivity. This would require a better understanding of the temporal and spatial regulation of ABA signaling, including the precise timing of hormone production and its localized effects on different tissues. To address these gaps, future research must integrate traditional genetic and biochemical approaches with emerging technologies such as single-cell transcriptomics, CRISPR-based gene editing, and machine learning. These tools will enable the detailed dissection of ABA signaling at the single-cell level, provide more precise modifications in ABA pathways, and model complex signaling interactions across the plant genome. For example, the ability to manipulate ABA signaling at the tissue-specific level in real-time could offer powerful new strategies for crop improvement, enhancing resilience to both abiotic and biotic stresses while minimizing trade-offs with plant growth.

## 2. Biosynthesis and Catabolism of ABA

Abscisic acid (ABA) plays a central role in regulating plant responses to environmental stress, including drought, salinity, and extreme temperatures. The biosynthesis and catabolism of ABA are critical for maintaining hormonal balance and ensuring plants’ adaptive responses to these stresses. While the biosynthetic pathways of ABA have been well-characterized, substantial gaps remain in our understanding of how this pathway integrates with other metabolic processes and how plants regulate ABA levels under varying environmental conditions. To better understand these processes, the following sections will explore the key pathways involved in ABA biosynthesis and the enzymes that drive these biochemical transformations.

### 2.1. Pathways of ABA Biosynthesis

Abscisic acid (ABA) biosynthesis is a tightly regulated process that begins in the plastids, where carotenoids such as 9-cis-violaxanthin and 9-cis-neoxanthin undergo oxidative cleavage by the enzyme 9-cis-epoxycarotenoid dioxygenase (NCED) [28,29]. This reaction produces xanthoxin, a pivotal intermediate that is subsequently transported to the cytosol, where it is converted into ABA through a series of enzymatic reactions. The transport of xanthoxin from the plastid to the cytosol remains an understudied aspect of the process, with recent studies suggesting the involvement of transporter proteins that could serve as regulatory bottlenecks [30,31].

NCED has been established as the rate-limiting enzyme in ABA biosynthesis, with its transcriptional regulation being a key point of control during stress responses [32,33]. The role of NCED is particularly evident under drought conditions, where rapid upregulation of its expression correlates with ABA accumulation [34]. Recent studies have revealed that this regulation is not only transcriptional but also involves post-transcriptional and post-translational mechanisms, including protein stability, phosphorylation, and feedback from ABA levels themselves [35].

In addition to NCED, novel enzymes such as ABA4 and LUT5 have been implicated in the early steps of the carotenoid pathway leading to ABA biosynthesis, suggesting that flux through the pathway may be more complex and plastic than previously assumed [36,37]. These findings expand the canonical view of the pathway and point toward alternative or branching routes that could fine-tune ABA levels in response to environmental conditions.

**Figure 2:**
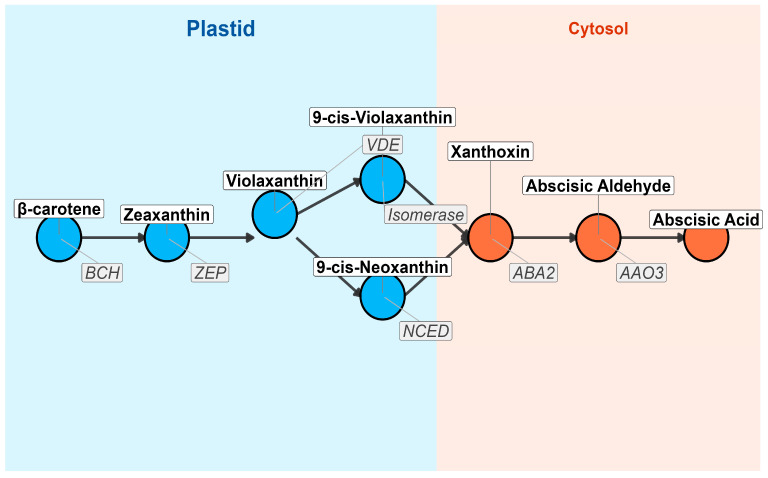
The biosynthetic pathway of ABA from β-carotene in the plastid to ABA in the cytosol is illustrated. Each node represents a key metabolite, while arrows indicate enzymatic conversions. The plastid compartment is shaded in blue, and the cytosol compartment is shaded in orange. Enzymes catalyzing each step are labeled adjacent to the arrows. The pathway begins with β-carotene, which undergoes sequential modifications via enzymes such as BCH (β-carotene hydroxylase), ZEP (Zeaxanthin Epoxidase), VDE (Violaxanthin De-epoxidase), Isomerase, NCED (9-cis-Epoxycarotenoid Dioxygenase), ABA2 (Short-chain Dehydrogenase/Reductase), and AAO3 (Abscisic Aldehyde Oxidase), ultimately producing ABA. This pathway plays a crucial role in plant stress responses, seed dormancy, and stomatal regulation.

However, beyond stress responses, the regulation of NCED activity during non-stress conditions remains less well understood. Emerging evidence indicates that other environmental cues, such as light, circadian rhythms, and nutrient availability, may also modulate NCED activity, suggesting a more dynamic regulation than previously thought [38,39]. Moreover, evolutionary analyses suggest that gene duplication and divergence of NCED isoforms may underlie species-specific stress adaptations, pointing to ABA biosynthesis as a flexible, evolving system rather than a static pathway [40]. This insight raises questions regarding the integration of these diverse signals into ABA biosynthesis and how plants balance growth and stress adaptation (Figure 2).

The subcellular transport of xanthoxin remains an open research question. While there is indirect evidence for the involvement of ATP-binding cassette (ABC) transporters, the identity of the specific transporters facilitating this process has not been conclusively determined [41,42]. Targeted research into transporter identification and functional analysis could provide critical insights into how ABA biosynthesis is spatially and temporally regulated within the cell.

### 2.2. Key Enzymes in ABA Biosynthesis

In addition to NCED, other enzymes play critical roles in ABA biosynthesis. Xanthoxin is converted into ABA-aldehyde by the short-chain dehydrogenase/reductase (SDR) enzyme ABA2, which is subsequently oxidized into active ABA by abscisic aldehyde oxidase (AAO3) [43]. While NCED serves as the primary regulatory checkpoint, ABA2 and AAO3 are essential for completing the biosynthetic pathway. Studies on these enzymes have revealed that their activity is modulated by post-translational modifications, such as phosphorylation and ubiquitination, adding a further layer of control over ABA production [44].

The role of ABA2 and AAO3 is particularly significant under conditions of prolonged or recurrent stress. While their upregulation in response to environmental stress has been documented, the spatiotemporal dynamics, how their activity varies across different tissues (spatial) and changes over time (temporal), are less well understood. Recent studies highlight that their activity is not uniform across tissues and varies with stress type and intensity, suggesting organ-specific regulation of ABA biosynthesis [45]. Comparative genomic studies have identified significant genetic diversity in ABA biosynthesis enzymes, with functional polymorphisms linked to stress tolerance in crop species [45]. These findings point to the possibility of breeding or engineering crops with enhanced ABA-mediated stress resilience.

Recent advances in proteomics and metabolomics have shed light on how post-translational modifications (PTMs) of these enzymes influence ABA biosynthesis. For instance, phosphorylation of NCED and ABA2 has been linked to changes in enzyme stability and catalytic activity [46]. Such regulatory mechanisms could play a vital role in plant adaptation to fluctuating stress conditions, warranting further investigation into the functional consequences of PTMs.

### 2.3. Regulation of ABA Catabolism and Homeostasis

ABA catabolism is a vital process that ensures the timely degradation of ABA once the stress signal subsides. This process is mediated by ABA 8′-hydroxylase enzymes encoded by CYP707A genes, which convert ABA into phaseic acid [47]. The upregulation of CYP707A during stress recovery highlights its role in restoring basal ABA levels after the stress response is complete [48]. Among the CYP707A family, CYP707A1 and CYP707A3 have been identified as key isoforms that are activated during rehydration after drought stress [49].

Recent studies suggest that the products of ABA catabolism, such as phaseic acid and dihydrophaseic acid, may have signaling roles of their own, possibly acting as secondary messengers in stress response pathways [50]. This raises questions about whether these intermediates should be viewed as mere byproducts or active participants in stress signaling. Further metabolomic and reverse genetics studies are required to elucidate the potential signaling roles of these compounds.

Catabolism plays a crucial role in recurrent stress cycles. Repeated stress exposure can induce “stress memory,” where prior stress enhances future responses by priming physiological and molecular mechanisms [51,52]. The role of ABA catabolism in this process remains underexplored; however, some studies suggest that rapid ABA degradation prevents prolonged accumulation, which could otherwise lead to growth inhibition or hypersensitivity to stress [53,54]. While direct experimental evidence remains limited, ABA catabolism is thought to contribute to the fine-tuning of stress responses by ensuring that ABA levels return to baseline after stress subsides. Understanding how plants coordinate ABA synthesis, signaling, and catabolism over repeated stress events could provide insights into how to engineer crops with improved long-term stress resilience.

## 3. ABA Signal Perception and Transduction

The perception and transduction of ABA signals enable plants to mount adaptive responses to environmental stress. ABA receptors of the PYR/PYL/RCAR family bind ABA, inhibiting protein phosphatases 2C (PP2C) and activating SNF1-related protein kinase 2 (SnRK2), which triggers downstream signaling events [55]. This cascade phosphorylates targets like ion channels, transcription factors, and metabolic enzymes, driving stress responses, including stomatal closure and gene expression changes.

Recent structural studies have provided detailed insights into receptor–ligand dynamics and the conformational changes that enable ABA perception. High-resolution cryo-EM and crystallographic analyses have revealed differences in binding affinities and regulatory mechanisms among PYR/PYL isoforms, raising questions about their functional specialization and redundancy under specific stress conditions [56,57]. Despite these advances, how the structural diversity of these receptors translates into differential signaling outputs in vivo remains poorly understood.

ABA not only mediates rapid stomatal closure in response to drought but also regulates stomatal development and density over longer periods. Under water-limiting conditions, ABA reduces stomatal initiation by inhibiting transcription factors such as SPEECHLESS (SPCH) through SnRK2 activation [58,59]. This allows plants to optimize water use by adjusting stomatal density and patterning. In addition to core components, recent transcriptomic and proteomic studies have identified novel regulators of ABA signaling through time-series analyses and genome-wide mutant screens. For instance, integrative omics approaches have revealed dynamic phosphorylation events and feedback regulation involving kinases such as RAF-like MAPKKKs, and newly identified modulators such as CARKs and ABI5-interacting proteins [60,61]. These studies highlight the complexity of ABA signal transduction and suggest that core signaling elements operate within broader, plastic networks that respond to specific stress intensities and durations.

Additionally, ABA-induced reactive oxygen species (ROS) enhance stomatal responses, interacting with signaling pathways like MAPK and modulating transcription factors such as ABI4 to coordinate ABA and gibberellin (GA) signaling [62]. However, the precise coordination between ROS, ABA, and hormone crosstalk under combinatorial stress remains unresolved, representing an important avenue for future investigation.

Environmental factors, such as elevated CO_2_ and drought stress, further influence ABA’s role in stomatal adaptation. Increased CO_2_ boosts ABA biosynthesis, reducing stomatal density over time [63]. Furthermore, desensitization mechanisms, such as N-glycosylation of SnRK2s, fine-tune prolonged ABA responses, preventing excessive suppression of stomatal development [64,65]. These processes ensure that ABA balances both immediate stress responses and long-term environmental acclimation.

### 3.1. ABA Receptors and Signal Transduction Components

The discovery of PYR/PYL/RCAR receptors has revolutionized our understanding of ABA signaling. Different isoforms of these receptors exhibit varying affinities for ABA and display diverse expression patterns across tissues and developmental stages [66,67]. This receptor diversity allows plants to fine-tune their responses to specific stress conditions. Research on receptor isoforms such as PYL4 and PYL5 highlights their enhanced activity under severe drought; however, further work is needed to establish how these isoforms interact with other stress signaling pathways.

Recent advances in structural biology have revealed the precise mechanisms by which ABA receptors bind to ABA and interact with PP2Cs. These structural insights provide a basis for developing synthetic chemicals that could modulate ABA signaling in crops [68]. This approach holds promise for enhancing drought resistance in agriculture, especially in arid regions.

### 3.2. ABA-Responsive Gene Expression

**Figure 3:**
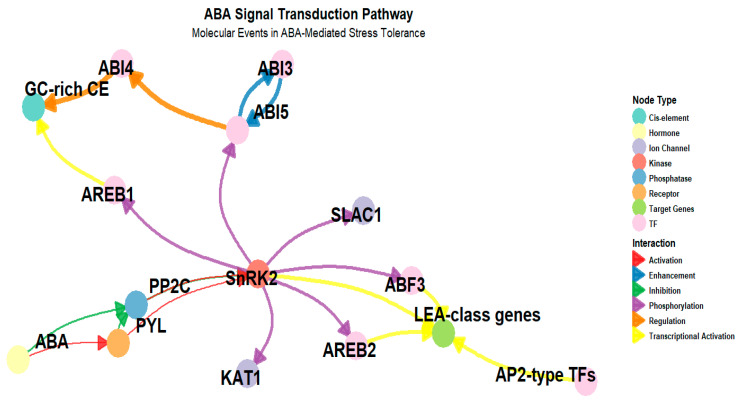
This figure illustrates the key molecular steps in ABA-responsive gene expression. ABA binds to PYL/RCAR receptors, leading to the inhibition of PP2C phosphatases and the activation of SnRK2 kinases. Activated SnRK2s phosphorylate ABF transcription factors, which then bind to ABRE and DRE elements in gene promoters, inducing the expression of stress-responsive genes such as RD29A and LEA-class genes. Node colors indicate molecule types, including hormones, receptors, kinases, phosphatases, and transcription factors, while edge colors and arrow shapes represent interaction types such as activation, inhibition, phosphorylation, and transcriptional activation. A negative feedback loop via PP2C helps regulate the timing and magnitude of ABA-induced gene expression.

The activation of ABA-responsive genes is mediated by ABA-responsive element (ABRE)-binding factors (ABFs), which bind to ABRE motifs in the promoters of stress-related genes [69]. This process is further modulated by secondary messengers such as calcium (Ca^2+^) and reactive oxygen species (ROS), which play crucial roles in amplifying and fine-tuning ABA signaling under different environmental conditions [70]. Ca^2+^ acts as a dynamic signaling molecule, translating ABA perception into downstream gene activation through Ca^2+^-dependent protein kinases, while ROS serve as both signaling intermediates and regulators of ABA sensitivity. The interplay between ROS and Ca^2+^ helps adjust the intensity and duration of ABA responses, ensuring plants can efficiently adapt to varying stress intensities. Recent studies suggest that ROS production is not merely a byproduct of stress but actively enhances ABA signaling efficiency, potentially creating feedback loops that reinforce stress adaptation [71,72] (Figure 3).

The role of epigenetic regulation in ABA-mediated stress responses is an emerging field. Histone modifications, such as H3K27me3, have been implicated in the priming of ABA-responsive genes, potentially influencing stress memory in plants [73]. Future research should focus on identifying specific histone-modifying enzymes that are responsive to ABA and exploring how their activity shapes the chromatin landscape during stress adaptation.

## 4. ABA as a Master Regulator of Plant Stress Responses and Development

Abscisic acid (ABA) plays a pivotal role in coordinating plant responses to environmental stresses and regulating key developmental processes. As a master regulator, ABA enables plants to adapt to fluctuating conditions by modulating water loss, controlling seed dormancy, and fine-tuning gene expression. Recent breakthroughs have advanced our understanding of how plants cope with prolonged ABA exposure. Studies by Lu et al. [74] have shown that prolonged ABA signaling leads to desensitization of the ABA response, which is a key aspect of how plants adapt to long-term stress. Their work suggests that N-glycosylation of SNF1-related protein kinase 2s (SnRK2s) plays a crucial role in regulating NADPH maintenance in peroxisomes during sustained ABA signaling, thereby modulating cellular responses to long-term stress. This finding highlights an important regulatory mechanism through which plants manage ABA levels and their signaling pathways over time, offering new avenues for enhancing stress resilience. Its role extends from inducing stomatal closure during drought stress to maintaining seed dormancy under unfavorable conditions and activating stress-responsive genes. Through these interconnected processes, ABA ensures plant survival and resilience in challenging environments. The following sections explore the specific roles of ABA in stomatal closure, seed dormancy and germination, and gene expression regulation, highlighting its central role in plant growth, development, and stress tolerance.

### 4.1. ABA in Stomatal Development and Closure

Stomatal regulation is a critical adaptation mechanism in plants, balancing photosynthetic efficiency with water conservation. Stomatal function is controlled on two levels: short-term responses, such as stomatal closure during drought stress, and long-term developmental adaptations, including stomatal density modulation. ABA plays a pivotal role in both processes, coordinating rapid responses to dehydration while also regulating stomatal development to optimize gas exchange under changing environmental conditions [75,76].

#### 4.1.1. ABA-Induced Stomatal Closure

Stomatal closure is one of the most immediate and vital responses to water deficit, preventing excessive water loss through transpiration. ABA accumulation under drought conditions triggers a well-characterized signaling cascade involving PYR/PYL/RCAR receptors, which bind ABA and inhibit protein phosphatase 2C (PP2C) activity. This inhibition releases SnRK2 kinases, which phosphorylate downstream targets involved in ion transport, such as SLAC1 (slow anion channel 1), resulting in the efflux of anions like chloride and malate from guard cells. The loss of these solutes reduces turgor pressure, leading to stomatal closure [66,77,78] (Table S1).

Beyond this core pathway, ROS serve as secondary messengers in ABA-induced stomatal closure. ABA activates NADPH oxidases (RBOHs), leading to ROS accumulation, which amplifies calcium (Ca^2+^) signaling and enhances the stomatal closure response [79,80]. Ca^2+^ oscillations further fine-tune ABA signaling, modulating stomatal aperture dynamics in response to fluctuating environmental conditions [81].

Despite extensive research on the mechanisms of stomatal closure, gaps remain in understanding the specificity of ROS and Ca^2+^ signaling in different environmental contexts. The interplay between ABA, ROS, and light/circadian rhythms remains underexplored, as does the role of systemic drought signals, such as mobile RNAs and peptides, in coordinating stomatal responses across tissues [82,83].

#### 4.1.2. ABA in Stomatal Development and Long-Term Adaptation

Beyond its role in short-term stomatal closure, ABA also modulates stomatal density and patterning to optimize water use efficiency over longer timescales. Stomatal development is governed by a transcriptional regulatory network involving SPEECHLESS (SPCH), MUTE, and FAMA, which drive the progression from meristemoid cells to mature guard cells [84,85]. ABA negatively regulates this pathway by suppressing SPCH, thereby restricting stomatal initiation under water-limiting conditions [86]. This suppression is mediated by the ABA-activated SnRK2 kinase family, which phosphorylates SPCH, marking it for degradation and halting stomatal lineage progression [87].

Additionally, ABA signaling influences the EPIDERMAL PATTERNING FACTOR (EPF) family, which modulates stomatal density through interactions with the ERECTA receptor kinase. Under drought stress, ABA induces EPF2 expression, restricting meristemoid proliferation and reducing stomatal density to enhance water conservation [88]. This mechanism highlights ABA’s role in fine-tuning stomatal patterning as an adaptive response to prolonged environmental challenges.

#### 4.1.3. ABA-ROS Crosstalk in Stomatal Development

Recent findings indicate that ROS, beyond their role in transient stomatal closure, are also integral to long-term stomatal development. ABA-induced ROS accumulation disrupts meristemoid division through MAPK signaling, particularly MPK3/MPK6, which phosphorylate SPCH and restrict stomatal formation under prolonged stress conditions [89,90,91]. Furthermore, ROS modulates the activity of ABA-INSENSITIVE 4 (ABI4), a transcription factor integrating ABA and gibberellin (GA) signaling. ABI4 represses GA biosynthesis genes, reinforcing the ABA-GA antagonistic interaction within stomatal lineage cells [92]. This coordination ensures that ABA-driven suppression of stomatal development under stress aligns with a broader reduction in vegetative growth, conserving resources under adverse conditions.

#### 4.1.4. Environmental Integration and Evolutionary Implications

The interaction between ABA, CO_2_ concentration, and drought stress has broad implications for plant adaptation. Increased atmospheric CO_2_ levels enhance ABA biosynthesis, which further suppresses stomatal formation, leading to long-term reductions in stomatal density. This pattern is reflected in fossil records, which indicate historical shifts in stomatal density as an adaptive response to fluctuating CO_2_ concentrations [93,94]. Prolonged ABA signaling, however, is subject to desensitization mechanisms that prevent excessive suppression of stomatal development. Lu et al. [74] recently demonstrated that N-glycosylation of SnRK2s in peroxisomes modulates NADPH homeostasis, attenuating ABA responses during extended exposure. Such regulatory fine-tuning underscores the complexity of ABA’s role in stomatal adaptation, extending beyond immediate drought responses to long-term environmental acclimation. Overexpression of the OsABA8ox3 gene, which is involved in ABA catabolism, has been shown to improve drought tolerance in rice (*Oryza sativa*) by enhancing stomatal closure and reducing water loss under drought conditions [95]. Similarly, the SnRK2 family of kinases, which are activated by ABA signaling, have been engineered to increase drought resistance in rice without compromising yield [96,97,98].

Understanding ABA’s role in stomatal regulation presents exciting opportunities for agricultural biotechnology. Manipulating ABA biosynthesis (e.g., through NCED3 overexpression) or its downstream effectors (e.g., SnRK2 kinases, MPK6) could enable precise control over stomatal density without compromising photosynthetic efficiency [99]. Additionally, the development of ABA analogs such as opabactin, which selectively activate ABA receptors, provides a promising strategy for engineering drought-resistant crops [100].

### 4.2. ABA and Gibberellins (GAs) in Seed Dormancy and Germination

Abscisic acid and GA are two key phytohormones that exhibit a well-documented antagonistic relationship in regulating seed dormancy and germination. Their interaction is essential for ensuring seeds remain dormant during unfavorable environmental conditions and only germinate when conditions are suitable for seedling establishment [101,102]. The molecular interplay between ABA and GA is fundamental in controlling the transition from dormancy to germination and is influenced by various environmental cues. Recent research has further elucidated the complex antagonistic interactions between ABA and gibberellins (GAs), highlighting the role of specific molecular modules. For instance, Xian et al. [92] identified the ABI4-RGL2 module as a crucial mediator of the ABA-GA crosstalk, where ABI4, a key ABA-responsive transcription factor, interacts with RGL2, a DELLA protein involved in GA signaling. This module serves as a double agent, coordinating the balance between ABA-induced seed dormancy and GA-promoted germination. The discovery of the ABI4-RGL2 interaction provides deeper insights into the regulatory network that maintains seed dormancy, especially under fluctuating environmental conditions [92]. Such breakthroughs advance our understanding of how plants finely tune their hormonal responses to optimize seed germination and dormancy based on environmental cues.

#### 4.2.1. ABA’s Role in Seed Dormancy

ABA plays a crucial role in maintaining seed dormancy by promoting metabolic inactivity and preventing premature germination. One of the key mechanisms through which ABA regulates dormancy is by activating a suite of transcription factors, including ABI3, ABI4, and ABI5 [103,104,105]. These transcription factors bind to specific promoter regions of genes involved in dormancy maintenance, such as DOG1 (Delay Of Germination 1) and RAB18 (responsive to ABA), leading to the repression of genes associated with germination [106,107]. This transcriptional regulation ensures that the seed remains in a dormant state until favorable environmental conditions, such as adequate moisture and temperature, are present.

ABA also inhibits gibberellin (GA) biosynthesis by suppressing the expression of key enzymes involved in GA production, such as GA3ox (gibberellin 3-oxidase), and promoting the expression of genes encoding enzymes responsible for GA inactivation [108,109]. In seeds, the reduction in GA levels prevents the activation of germination processes, including the breakdown of seed reserves and the growth of the embryo.

#### 4.2.2. Gibberellin (GA) and Seed Germination

In contrast, GA is essential for seed germination. Upon exposure to favorable environmental conditions, GA levels rise, which promotes the breakdown of DELLA proteins, negative regulators of GA signaling. DELLA proteins interact with transcription factors in the GA signaling pathway and inhibit the expression of genes required for germination [110,111]. The degradation of DELLA proteins removes this inhibition, allowing the activation of genes that promote seed germination, such as those involved in endosperm mobilization (e.g., α-amylase). The balance between ABA and GA is therefore pivotal in regulating the timing of germination.

#### 4.2.3. Interaction Between ABA and GA

The antagonism between ABA and GA in seed dormancy and germination is a dynamic process, with environmental factors such as temperature, light, and water availability modulating their balance. Under drought or cold stress, ABA levels increase, which in turn promotes dormancy and prevents germination [112]. Conversely, when conditions are favorable for germination, GA levels rise, and ABA levels decrease, leading to the activation of germination processes [113,114].

The molecular regulation of the ABA-GA balance involves the interplay of various signaling pathways and transcription factors. For example, DELLA proteins, which mediate GA signaling, also participate in ABA signaling. DELLA proteins can physically interact with ABA receptors, such as PYR1 (Pyrabactin resistance 1), influencing ABA signal transduction [115,116]. This makes DELLA proteins key convergence points for ABA and GA signaling, facilitating the coordination of dormancy and germination processes.

#### 4.2.4. Environmental Modulation of ABA and GA Interaction

The interaction between ABA and GA in regulating seed dormancy is strongly influenced by environmental factors. Temperature, in particular, plays a significant role in modulating this balance. Cold stress induces ABA accumulation, promoting dormancy, while higher temperatures typically favor the synthesis of gibberellins, leading to germination [117]. Moreover, drought conditions also favor ABA accumulation, which reinforces dormancy mechanisms in seeds to prevent germination under water-limited conditions [118].

Recent research has focused on the environmental modulation of ABA-GA interactions in specific crop species. For example, in wheat and rice, which are important crop species, researchers have found that exposure to drought stress results in an increase in ABA content, which suppresses GA biosynthesis, thus maintaining seed dormancy [119]. Understanding how ABA and GA interact under variable environmental conditions will help optimize seed performance, particularly in stress-prone agricultural regions.

#### 4.2.5. Practical Implications: Seed Priming and Crop Resilience

The manipulation of the ABA-GA balance holds great promise for improving agricultural practices, especially through seed priming techniques. Seed priming involves pre-treating seeds with a solution that enhances their stress tolerance and accelerates their germination under controlled conditions [120,121]. This process can be regulated by modulating the ABA-GA balance to induce a more favorable germination process. For instance, priming seeds with ABA can enhance dormancy under stress, while GA application can promote germination when the environmental conditions are suitable [122]. Thus, understanding the regulatory mechanisms between ABA and GA in seed dormancy is essential for developing stress-resilient crop varieties that can withstand fluctuating environmental conditions.

### 4.3. Gene Expression Regulation

The regulation of gene expression by ABA is a cornerstone of the plant’s ability to adapt to stress. ABA induces the expression of a variety of genes that help plants respond to environmental stressors. The molecular mechanisms involved in ABA-regulated gene expression are complex, involving transcription factors that bind to ABA-responsive elements (ABREs) and modulate the expression of stress-responsive genes.

One key family of transcription factors involved in ABA-induced gene expression is the ABF (ABRE-binding factors)/AREB family, which is activated downstream of SnRK2 kinases. These transcription factors bind to ABRE motifs in gene promoters, leading to the activation of stress-related genes, including those involved in antioxidant defense, osmotic regulation, and stomatal control [123,124]. In addition to these transcriptional regulators, ABA also affects epigenetic modifications, such as histone acetylation, which can alter chromatin structure and influence gene expression [125]. Moreover, post-transcriptional mechanisms, including the regulation of microRNAs like miR159, add an additional layer of complexity to the regulation of ABA responses.

While the core mechanisms of ABA-induced gene expression are well-documented, several important gaps remain in our understanding. For example, the spatial and temporal dynamics of ABA-regulated transcription across different tissues are poorly understood. ABA’s effects on gene expression likely vary between different cell types, tissues, and developmental stages, and these variations may be critical for fine-tuning stress responses. A more detailed understanding of how ABA regulates gene expression in a tissue-specific manner could provide insights into optimizing stress tolerance in plants. Furthermore, the contribution of non-coding RNAs and alternative splicing in modulating ABA responses remains underexplored. Non-coding RNAs, including long non-coding RNAs (lncRNAs) and microRNAs, have been shown to play important roles in gene regulation; however, their involvement in ABA signaling pathways has not been fully characterized. Additionally, alternative splicing of ABA-responsive genes could provide another level of regulatory complexity, allowing plants to generate different protein isoforms in response to varying stress conditions. Addressing these gaps in research could uncover new regulatory mechanisms that could be harnessed to improve plant resilience.

## 5. Crosstalk Between ABA and Other Signaling Pathways

The role of ABA as a central regulator of plant stress responses is reinforced by its extensive interactions with other signaling pathways. Rather than acting in isolation, ABA integrates signals from both hormonal and environmental stimuli to modulate plant adaptation under stress [126]. This crosstalk is critical for fine-tuning stress responses, optimizing growth, and balancing survival and development. While past research has provided foundational insights into the nature of these interactions, recent studies have focused on the molecular mechanisms governing ABA’s crosstalk with other signaling pathways, particularly in the context of climate change and the demand for more resilient crops [127,128].

Advancements in multi-omics approaches, including transcriptomics, metabolomics, and proteomics, have provided a holistic view of how ABA interacts with other phytohormones and environmental cues. These integrative approaches are essential for unraveling the spatiotemporal aspects of ABA signaling and its dynamic interplay with other signals. Additionally, computational modeling of signaling networks is offering new insights into how plants prioritize and coordinate their responses to multiple stressors simultaneously [129,130].

### 5.1. Interaction with Other Phytohormones

- How does ABA coordinate with other plant hormones to balance growth and stress responses in changing environments?

ABA’s interaction with other phytohormones, such as gibberellins (GAs), auxins, and ethylene, plays a pivotal role in shaping plant development and stress responses. These interactions, which can be antagonistic, synergistic, or context-dependent, highlight the dynamic and multifaceted nature of hormonal crosstalk. Recent studies emphasize that hormonal crosstalk is critical in determining stress outcomes in plants exposed to complex and fluctuating environments [131].

#### 5.1.1. ABA and Gibberellins (GAs)

- How does the antagonism between ABA and GA fine-tune seed dormancy and germination under stress conditions?

The interplay between ABA and GA not only governs seed dormancy and germination but also adapts these developmental transitions to environmental stress. Under stress conditions, such as drought, cold, or salinity, the hormonal balance shifts toward ABA dominance, enhancing seed dormancy as a survival strategy. Conversely, GA-driven germination is promoted under favorable conditions, ensuring optimal seedling establishment.

This hormonal crosstalk becomes especially relevant in stress-adaptive agriculture. For instance, seed priming techniques that modulate ABA and GA levels have been used to improve crop emergence under suboptimal environments. Moreover, understanding how DELLA proteins integrate ABA-GA signaling under stress provides opportunities to genetically fine-tune germination timing and stress resilience. Future research into how abiotic stressors dynamically reshape the ABA-GA regulatory network may lead to targeted strategies for crop improvement in climate-unstable regions.

#### 5.1.2. ABA and Auxins

- How does the interplay between ABA and auxins shape root system architecture in response to different types of stress?

The crosstalk between ABA and auxins is vital for regulating root growth and architecture, especially under water-deficit stress. While ABA generally inhibits root elongation to reduce water loss, auxins promote root growth and branching. Under drought conditions, ABA restricts the transport of auxins via PIN-type auxin efflux carriers, effectively reducing root elongation [132]. However, in other stress contexts, such as salt stress, ABA and auxin signaling can work together to adjust root architecture, promoting lateral root formation for better nutrient acquisition [52]. Genetic engineering of SlAREB1 (an ABA-responsive element-binding factor) in tomato (*Solanum lycopersicum*) led to enhanced salt tolerance by upregulating the expression of stress-responsive genes and improving osmotic balance [133,134]. Transgenic tomatoes overexpressing SlAREB1 exhibited higher fruit yield under salinity stress conditions.

New evidence from studies on model plants like *Arabidopsis thaliana* reveals that the balance between ABA and auxins is context-dependent, influenced by stress type, intensity, and duration [135]. Researchers have identified several transcription factors, such as ABI3 and ARF2, that mediate the ABA-auxin interaction, indicating the presence of a tightly regulated feedback loop.

#### 5.1.3. ABA and Ethylene

- In what ways do ABA and ethylene antagonize or cooperate to regulate stomatal responses and stress signaling?

The relationship between ABA and ethylene is complex, as the two hormones often exhibit antagonistic behavior during stress responses. Ethylene suppresses ABA-induced stomatal closure, prioritizing gaseous exchange and photosynthesis [136,137]. This antagonism is critical for plants facing multiple stresses, as ABA promotes stomatal closure to reduce water loss, while ethylene maintains stomatal opening for gas exchange and photosynthesis.

**Figure 4:**
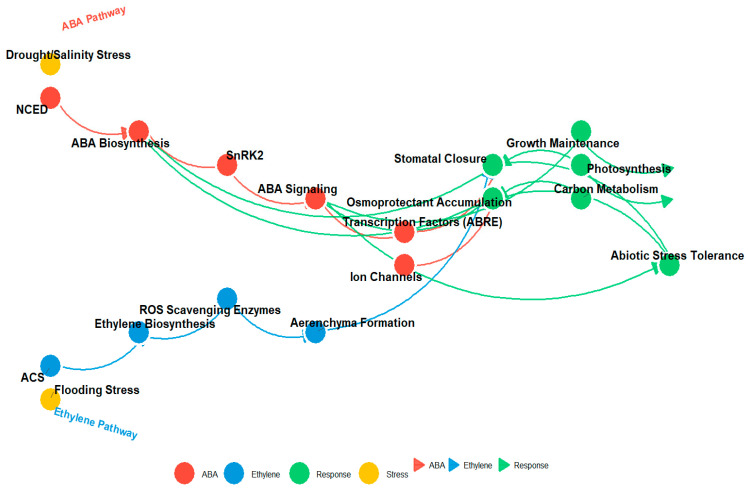
This diagram illustrates the interplay between the ABA (red) and ethylene (blue) signaling pathways and their downstream effects on plant responses to stress. Key components include NCED and SnRK2 in the ABA pathway, ACS in the ethylene pathway, and various physiological responses such as stomatal closure, osmoprotectant accumulation, and stress tolerance. Arrows represent molecular interactions, with node colors denoting pathway components (ABA, ethylene, response, and stress) and interaction strength indicated by arrow thickness.

Recent findings suggest that ABA-ethylene crosstalk is mediated by reactive oxygen species (ROS) and the phosphorylation of SnRK2 kinases, which are key players in ABA signaling [138]. Moreover, ethylene appears to influence the expression of ABA biosynthesis genes like NCED3, thereby creating feedback loops that control stomatal responses to environmental cues (Figure 4).

### 5.2. ABA Crosstalk with Nutrient Signaling in Plant Development and Stress Responses

Plant growth and development are governed by intricate signaling networks that integrate environmental cues, hormone signaling, and nutrient availability. ABA, primarily recognized for its role in seed dormancy and stress adaptation, is also closely linked to nutrient signaling pathways, influencing how plants allocate resources under varying environmental conditions. However, the molecular mechanisms underlying ABA-nutrient interactions remain underexplored.

#### 5.2.1. ABA and Nitrogen Signaling

Nitrogen availability is a key determinant of plant growth, and recent studies suggest that ABA interacts with nitrogen-signaling pathways to regulate root development, stomatal function, and stress responses. Under nitrogen deficiency, ABA levels increase, leading to modifications in root architecture, such as enhanced lateral root formation and reduced primary root elongation [139]. This interaction involves nitrate signaling components like NLP7 and the nitrate transporter NRT1.1, which are crucial in regulating nitrogen uptake efficiency [140]. Additionally, ABA modulates the expression of nitrate transporters, including NRT2.1, optimizing nitrogen acquisition under drought conditions [141]. The trade-off between stress response and growth is also evident, as ABA inhibits excessive vegetative growth under nitrogen-limited environments, prioritizing resource allocation toward stress adaptation [142,143].

#### 5.2.2. ABA and Phosphorus Signaling

Phosphorus availability similarly influences ABA accumulation, particularly in root system remodeling and metabolic adjustments. Under phosphorus deficiency, ABA promotes lateral root growth and root hair formation, facilitating phosphorus uptake [144]. There is also evidence of an interaction between ABA and sugar metabolism under low phosphorus conditions, where ABA regulates photosynthate partitioning and carbohydrate metabolism, ensuring energy balance under stress [145]. Recent findings indicate that ABA also modulates the expression of phosphate transporters, such as PHT1, linking phosphorus availability to ABA-mediated stress responses [146,147].

#### 5.2.3. ABA and Other Nutrient Pathways

Beyond nitrogen and phosphorus, ABA interacts with other nutrient pathways, including sulfur and potassium signaling. ABA influences sulfur metabolism by modulating glutathione biosynthesis, a key antioxidant defense mechanism protecting plants from oxidative stress during abiotic challenges [148,149]. Additionally, ABA plays a role in potassium transport, regulating ion channels such as SLAC1 and KUP transporters, which are essential for maintaining stomatal conductance and drought tolerance [150]. These interactions highlight the diverse roles of ABA in nutrient signaling beyond its traditional functions in seed dormancy and abiotic stress responses.

With advances in omics approaches, including transcriptomics, metabolomics, and phosphoproteomics, researchers are beginning to explore ABA’s role in nutrient signaling more comprehensively. CRISPR-based functional genomics enables targeted manipulation of ABA-related nutrient signaling regulators, while metabolomic profiling helps identify novel metabolites linking ABA and nutrient availability under stress conditions [151]. AI-driven modeling further enhances our ability to predict plant responses under different ABA-nutrient interaction scenarios, providing new insights into optimizing crop performance [152]. While ABA has been extensively studied in stress physiology, its interaction with nutrient signaling pathways remains an emerging area of research. Understanding how ABA regulates nutrient uptake, metabolism, and resource allocation could provide valuable insights into designing nutrient-efficient, stress-resilient crops in response to climate variability. Future research should focus on dissecting these interactions at molecular, cellular, and whole-plant levels using cutting-edge technologies to unlock new agricultural applications.

### 5.3. ABA and Environmental Signals

**Figure 5:**
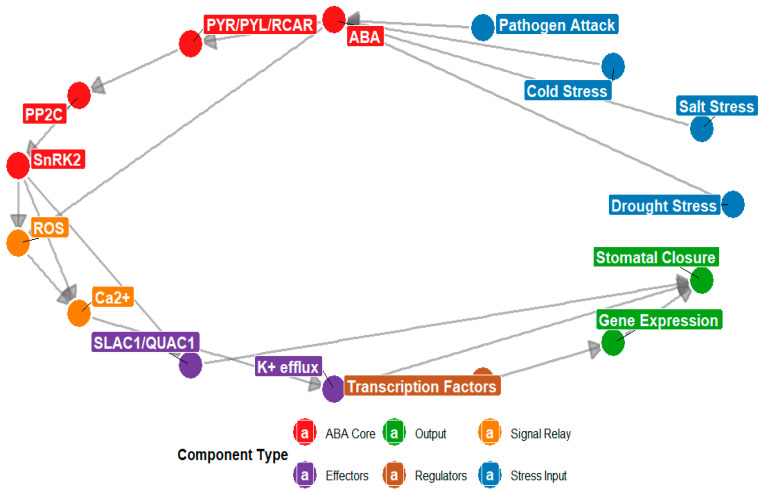
The diagram illustrates the ABA signaling network under environmental stress. Node colors represent functional categories: red indicates ABA, PYR/PYL/RCAR, PP2C, and SnRK2 (hormone core components); blue represents drought, cold, salt, and pathogen stress signals; orange denotes ROS and Ca^2+^; green indicates stomatal closure and gene expression (stress responses); rust represents transcription factors; and purple indicates SLAC1/QUAC1 and K^+^ efflux. Directed arrows indicate regulatory interactions such as activation or signal transduction, highlighting ABA’s central role in integrating environmental cues to drive stomatal closure and gene expression.

In addition to its interactions with phytohormones, ABA is a crucial mediator of plant responses to environmental factors such as light, temperature, and circadian rhythms. These interactions ensure that plants can anticipate and respond to daily and seasonal fluctuations in environmental conditions (Figure 5).

#### 5.3.1. ABA and Light Signaling

Light plays a fundamental role in regulating ABA biosynthesis, accumulation, and signaling pathways. Phytochromes and cryptochromes, acting as photoreceptors, influence ABA responses by controlling the expression of key biosynthesis genes such as NCED3, which encodes a rate-limiting enzyme in ABA production [153]. Light intensity and photoperiod directly impact ABA levels, particularly in guard cells, where ABA governs stomatal dynamics. Under high light conditions, reduced ABA accumulation promotes stomatal opening to facilitate CO_2_ uptake for photosynthesis, whereas low light or darkness enhances ABA-induced stomatal closure to minimize water loss.

Recent studies have revealed that the circadian clock integrates light signals with ABA signaling, modulating the timing of ABA-induced stomatal closure [154,155]. This synchronization ensures that stomatal responses align with predictable environmental changes, such as reduced transpiration demand at night or seasonal drought conditions. By fine-tuning ABA levels in response to light cues, plants optimize photosynthesis, water use efficiency, and stress adaptation.

#### 5.3.2. ABA and Temperature Stress

Temperature extremes, particularly cold stress, significantly impact ABA biosynthesis and signaling. ABA levels increase under cold stress, leading to the induction of cold-responsive (COR) genes, which enhance plant freezing tolerance [156]. The ABA-dependent cold response is linked to the activity of CBF/DREB transcription factors, which activate stress-protective genes. Overexpression of the CBF/DREB1 (C-repeat binding factor) gene family, which is activated by ABA under low-temperature stress, improved cold tolerance in *Arabidopsis* by increasing the accumulation of osmoprotectants and antioxidants [157].

Recent work suggests that temperature-induced ABA signaling is not an isolated event but part of a broader stress response network involving reactive oxygen species (ROS) and calcium (Ca^2+^) signaling [158]. Furthermore, ABA interacts with other hormones, such as ethylene and salicylic acid (SA), to fine-tune plant responses to both abiotic and biotic stressors. This hormonal crosstalk highlights the complexity of stress adaptation, where ABA serves as a central regulator integrating multiple environmental signals.

### 5.4. Biotic Stress Responses: Linking Environmental Stress to Plant Immunity

While ABA is primarily recognized for its role in adapting to environmental stresses like drought, light fluctuations, and temperature extremes, it also plays a critical role in biotic stress responses. The same signaling components that mediate ABA responses to abiotic stress, such as ROS, calcium signaling, and hormonal interactions, also influence plant immune responses to pathogens and herbivores.

#### ABA and Pathogen Defense

ABA has a dual role in pathogen defense. On one hand, it strengthens physical barriers by promoting callose deposition and enhancing ROS production, both of which restrict pathogen entry and spread. On the other hand, prolonged ABA signaling can suppress the salicylic acid (SA) pathway, which is crucial for defending against biotrophic pathogens [159,160]. This suppression occurs via several mechanisms: ABA has been shown to interfere with the expression and activity of key components in the SA signaling pathway, particularly the NPR1 (Nonexpressor of Pathogenesis-Related genes 1) protein. NPR1 is a central regulator of the SA-mediated immune response, and studies suggest that ABA can reduce NPR1 accumulation or its activity, thereby inhibiting the SA-induced defense response [161]. Additionally, the interaction between ABA and SA signaling pathways is often described as antagonistic, especially in the context of defense responses. In some cases, ABA may enhance the expression of jasmonic acid (JA)-related genes while suppressing SA-responsive genes. The balance between these two signaling pathways is crucial for determining whether the plant favors a growth or defense response, and prolonged ABA signaling may tip the scale towards growth suppression rather than immune activation [162,163]. Specific transcription factors, such as ABI5 (ABA-insensitive 5), activated under prolonged ABA signaling, have been shown to downregulate SA-responsive genes, further reducing the plant’s ability to mount a robust immune response against biotrophic pathogens [164].

This antagonistic ABA-SA interaction illustrates the trade-offs plants must navigate between defense and growth. While investment in defense mechanisms enhances survival under pathogen attack, excessive defense signaling may reduce growth and development. ABA-mediated regulation helps plants dynamically adjust this balance based on environmental conditions, ensuring optimal resource allocation between survival and productivity (Figure 5).

Emerging research suggests that ABA-mediated responses to pathogens are context-dependent. For example, ABA enhances resistance to necrotrophic pathogens but may increase susceptibility to biotrophic pathogens. This dual role has significant implications for breeding crops with enhanced disease resistance.

### 5.5. ABA Mimetics and Agricultural Applications

The potential of ABA analogs, such as opabactin, in improving crop stress resilience and productivity under adverse environmental conditions has gained significant attention. ABA mimetics, designed to mimic the natural effects of ABA signaling, could provide a promising tool for enhancing plant stress tolerance, particularly in agriculture where water scarcity and extreme weather events are becoming increasingly prevalent [165,166]. ABA analogs work by activating the ABA signaling pathway, which regulates various stress-responsive genes involved in stomatal closure, osmotic adjustment, and antioxidant defense mechanisms. The introduction of ABA mimetics can help plants better cope with abiotic stresses such as drought, salinity, and extreme temperatures by modulating these pathways even under non-optimal conditions [167,168].

Opabactin, an ABA analog developed by Vaidya et al. [99], has been shown to significantly improve drought tolerance in crop species by enhancing ABA-like responses without affecting overall plant growth. This particular compound interacts with the same molecular targets as endogenous ABA but provides the advantage of being more stable and less prone to degradation. This stability enhances its potential as a tool for field applications, where it can be sprayed on plants to improve stress resistance without the need for genetic modification [100].

#### 5.5.1. Underlying Mechanisms of ABA Mimetics

ABA mimetics like opabactin trigger similar molecular mechanisms to natural ABA, including the following:

Stomatal closure: ABA mimetics induce stomatal closure under drought conditions, reducing water loss and conserving soil moisture, a crucial factor in drought resistance.

Gene expression modulation: ABA analogs upregulate genes involved in osmotic regulation and stress tolerance, such as RD29A and P5CS, leading to enhanced cellular water retention and protection against oxidative damage.

Antioxidant response: ABA mimetics also activate antioxidant defense mechanisms, reducing oxidative stress during abiotic stress events.

#### 5.5.2. Translational Potential and Challenges

The application of ABA mimetics in agriculture could revolutionize the management of crop stress, particularly in arid regions. However, several challenges remain, including the following:

Field Stability: Ensuring that ABA mimetics retain their activity under varying environmental conditions, such as UV radiation, temperature fluctuations, and humidity, is essential for their practical use.

Cost and Production: Developing cost-effective synthesis methods for ABA analogs and ensuring that they are affordable for widespread use in agriculture is a key consideration.

Regulatory Approval: As with any new agricultural product, obtaining regulatory approval for the use of ABA mimetics in food crops will require extensive testing for safety and efficacy.

Recent studies also suggest that the use of ABA mimetics in combination with other agronomic practices, such as soil moisture management and crop breeding for drought tolerance, could provide synergistic effects, further enhancing agricultural productivity under stress conditions (e.g., water-scarce environments) [169,170]. The application of ABA mimetics such as opabactin holds great promise for improving crop resilience to abiotic stress, particularly in the context of global climate change. While further research and development are needed to optimize their use in agricultural practices, the translational potential of these compounds could offer significant benefits for sustainable crop production in the face of increasing environmental challenges.

## 6. Recent Advances in ABA Research

Recent advances in ABA research have been propelled by technological innovations in genetic manipulation, omics approaches, and cutting-edge tools. These developments have expanded our understanding of ABA’s role in plant stress responses, yet several critical gaps persist that need further investigation to optimize ABA’s potential in agricultural applications.

In terms of genetic and genomic insights, genome-editing tools like CRISPR/Cas9 have become instrumental in dissecting ABA signaling pathways. For example, targeted mutations in genes encoding ABA receptors (PYR/PYL) and downstream signaling proteins, such as SnRK2 kinases, have illuminated their roles in regulating stress responses. Additionally, classical ABA mutants, such as aba1, which is deficient in ABA biosynthesis, and snrk2 mutants, have provided valuable information about the redundancy and specificity of ABA signaling components [171,172]. However, while these studies have advanced our understanding of ABA signaling in model plants, their application in polyploid crops with complex genomes remains a significant challenge. The need for more refined tools to manipulate ABA signaling in crop species, alongside strategies to overcome regulatory barriers in genetic engineering, remains a critical area for further research.

Another significant advancement in ABA research comes from omics technologies, which have transformed the way we analyze the plant’s response to stress [173]. High-throughput transcriptomic studies have identified thousands of genes responsive to ABA under abiotic stress conditions, such as drought, salinity, and temperature extremes. These studies have highlighted the complex regulatory networks that govern ABA’s effects on gene expression, including those involved in ROS detoxification, osmotic regulation, and secondary metabolite production [174,175,176]. Similarly, proteomic and metabolomic studies have pinpointed proteins and metabolites that are modulated by ABA, contributing to the plant’s ability to adapt to stress [177]. However, despite these advancements, the integration of data from transcriptomics, proteomics, and metabolomics remains fragmented, with much of the existing research focusing on individual datasets. Future efforts should aim to combine these data streams to obtain a holistic view of ABA’s role in plant stress responses, enabling a more comprehensive understanding of its molecular and physiological mechanisms.

The use of emerging tools and techniques has also opened new frontiers in ABA research. Techniques like live-cell imaging have provided real-time insights into ABA dynamics, allowing researchers to track hormone movement and its effects on cellular processes like stomatal closure [178,179]. Fluorescent sensors for ABA, such as ABAleons, have made it possible to visualize the distribution and concentration of ABA within plant tissues. Additionally, advancements in single-cell transcriptomics are allowing scientists to investigate ABA’s impact on gene expression at the single-cell level, revealing how different cell types within a tissue respond to ABA [180,181]. This is particularly important in tissues with high cell-type heterogeneity, such as roots or leaf epidermal cells. Moreover, the growing use of machine learning and computational tools is enabling the prediction of ABA-responsive genes and the modeling of their signaling networks. These innovations have the potential to revolutionize ABA research; however, their full application is still in its early stages. The lack of large, comprehensive datasets, as well as the complexity of ABA’s interactions with other signaling pathways, presents challenges for the broader adoption of machine learning approaches [182,183].

Recent advancements in genetic tools, omics approaches, and emerging technologies have significantly deepened our understanding of ABA’s role in stress tolerance. However, there are still critical gaps, particularly in integrating multiple datasets from omics studies and applying these insights to crops. The continued refinement of genetic manipulation techniques and the expansion of interdisciplinary research will be essential in harnessing ABA’s full potential for improving plant stress resilience, particularly in the context of global climate change and increasing environmental challenges (Table S2).

## 7. Conclusions and Perspectives

Abscisic acid plays a pivotal role in regulating plant responses to abiotic stresses, such as drought, salinity, and extreme temperatures, while also influencing key growth and developmental processes. While significant progress has been made in understanding ABA’s molecular mechanisms, there are still considerable gaps that hinder the full application of this knowledge to improve stress tolerance in crops. One of the primary challenges is understanding how ABA functions under conditions of multiple combinatorial stresses, such as drought in combination with high temperature or salinity, which plants frequently experience in natural and agricultural settings. Although research has identified critical regulatory genes and pathways involved in ABA signaling, the interaction between ABA and other phytohormones, environmental signals, and biotic stress responses remains inadequately characterized.

To address these issues, it is essential for future research to move beyond model organisms and focus on integrating insights into crop species that face real-world challenges in diverse and changing environments. One promising approach could be the application of advanced “omics” technologies, which provide a more holistic view of ABA-mediated responses across the plant’s genome, proteome, and metabolome. Additionally, leveraging emerging tools such as CRISPR-Cas9 for precise gene editing could allow for targeted manipulation of ABA signaling pathways in crops, facilitating the development of stress-resilient varieties with improved productivity.

Furthermore, the role of ABA in regulating plant responses to biotic stress, such as pathogen resistance, has been underexplored and deserves more attention, particularly given the increasing threat of disease in stressed plants. A deeper understanding of ABA’s interplay with the plant immune system could open new avenues for improving resistance to both biotrophic and necrotrophic pathogens.

In conclusion, future efforts should prioritize a systems-level understanding of ABA’s role in plant adaptation to multiple environmental stresses. By addressing these research gaps, integrating knowledge across disciplines, and applying cutting-edge technologies, we can unlock the potential of ABA to enhance crop resilience, providing critical solutions for agriculture in the face of global climate change.

## Supplementary Materials

The following supporting information can be downloaded at: https://www.mdpi.com/article/10.3390/ijms26167872/s1.
