# Supplementary material for: Molecular Insights into ABA-Mediated Regulation of Stress Tolerance and Development in Plants
Source: Int J Mol Sci. 2025 Aug 15;26(16):7872. doi: 10.3390/ijms26167872 (PMC12387064; doi:10.3390/ijms26167872)
Supplement: Supplementary file 1 [file ijms-26-07872-s001.zip › ijms-3744748-supplementary.pdf]

Table S1. Role of Abscisic Acid as a Master Regulator of Plant Stress Responses and Development.

Section	Biological Role	Molecular Mechanism	Key Signaling Components	Research Gaps and Future Directions	References
ABA as a Master Regulator	ABA regulates plant adaptation to stress, balancing growth, development, and survival. It mediates stomatal closure, seed dormancy, and stress-induced gene expression.	- ABA binds to PYR/PYL/RCAR receptors, inhibiting PP2C phosphatase.<br>- Inhibition of PP2C activates SnRK2 kinases, which phosphorylate downstream targets.<br>- Phosphorylation of key transcription factors (e.g., ABI3, ABI4, ABI5) regulates stress-responsive gene expression.	**Core Pathway:** PYR/PYL/RCAR → PP2C → SnRK2 → Downstream targets (ion channels, transcription factors).<br>**Key Effectors:** Ion channels (SLAC1, KAT1) and transcription factors (ABI3, ABI4, ABI5) control water loss and seed dormancy.	- Elucidate how ABA signaling integrates with other environmental factors (e.g., light, humidity, circadian rhythms).<br>- Investigate cross-talk with systemic long-distance signals, including RNA and peptide signaling.	Lu et al. (2024); Vaidya et al. (2022); Cutler et al. (2010); Finkelstein et al. (2002)
Stomatal Closure	ABA-mediated stomatal closure prevents water loss during drought, ensuring water-use efficiency.	- **Perception:** ABA binds to PYR/PYL/RCAR receptors.<br>- **Signal Transduction:** Receptor binding inhibits PP2C, leading to SnRK2 kinase activation.<br>- **Response:** SnRK2 phosphorylates ion channels (SLAC1) to promote ion efflux, reducing turgor pressure and inducing stomatal closure.<br>- **Secondary Signals:** Reactive oxygen species (ROS) and calcium ions (Ca^{2+}) serve as secondary messengers. ROS production via RBOH NADPH oxidases amplifies the ABA signal.	**Receptors:** PYR/PYL/RCAR.<br>**Kinases/Phosphatases:** PP2C (negative regulator) and SnRK2 (positive regulator).<br>**Ion Channels:** SLAC1 (anion channel) and KAT1 (K+ channel) control ion efflux.<br>**Secondary Messengers:** ROS (NADPH oxidase) and Ca^{2+} (calcium oscillations) amplify the signal.	- Determine how ROS specificity is achieved under diverse environmental conditions (light, humidity, temperature).<br>- Investigate the role of circadian rhythms in ABA-driven stomatal closure.<br>- Identify mobile signals (e.g., RNA, peptides) that coordinate stomatal closure across plant tissues.	Geiger et al. (2011); Zhang et al. (2022); Schroeder et al. (2001); Hossain et al. (2021)
Seed Dormancy and Germination	ABA maintains seed dormancy and prevents premature germination, ensuring germination occurs	- **Perception:** ABA binds to PYR/PYL/RCAR receptors, triggering signal transduction.<br>- **Signal Transduction:** PP2C inhibition activates SnRK2,	**Receptors:** PYR/PYL/RCAR.<br>**Transcription Factors:** ABI3, ABI4, and ABI5 (core regulators of dormancy-related gene expression).	- Clarify the molecular mechanisms of thermoinhibition (temperature-	Lee et al. (2010); Nambara &

	under favorable conditions.	which phosphorylates transcription factors ABI3, ABI4, and ABI5.<br>- **Response:** These transcription factors activate dormancy-related genes, while ABA suppresses gibberellin (GA) biosynthesis, inhibiting germination.<br>- **Cross-talk:** The balance of ABA (dormancy inducer) and GA (germination promoter) controls seed fate. DELLA proteins stabilize dormancy by repressing GA signaling.	**Cross-talk Elements:** DELLA proteins negatively regulate GA signaling.<br>**Biosynthesis Pathways:** ABA biosynthesis and GA biosynthesis are antagonistic.	induced dormancy) and its interaction with ABA pathways.<br>- Elucidate the role of ABA in seed heteromorphism (seeds with distinct dormancy traits within one plant).<br>- Investigate crop-specific mechanisms of ABA-induced seed dormancy, especially in wheat, rice, and maize.	Marion-Poll (2005); Finkelstein (2013); Kucera et al. (2005)

Table S2. Recent Advances in ABA Research

Research Area	Advancement/Breakthrough	Key Tools and Technologies	Impact on ABA Research	Current Challenges and Research Gaps	References
Genetic and Genomic Insights	- Identification of key ABA signaling components (PYR/PYL, PP2C, SnRK2) via CRISPR/Cas9 genome editing.<br>- Development of mutant lines (e.g., *aba1* and *snrk2*) to elucidate gene-specific roles.	**CRISPR/Cas9 Genome Editing** for targeted gene disruption.<br>**Classical Mutants** like *aba1* and *snrk2* for functional analysis.	- Enhanced understanding of ABA receptor-ligand specificity.<br>- Identification of redundancy and specificity of ABA components.<br>- New insights into the roles of SnRK2 kinases and their interaction with PP2C.	- Difficulty in applying CRISPR/Cas9 to polyploid crops (e.g., wheat, sugarcane) due to genome complexity.<br>- Regulatory hurdles in using CRISPR-edited plants for commercial agriculture.<br>- Limited studies on crop-specific ABA signaling compared to model plants (e.g., *Arabidopsis*).	Zhang et al. (2023); Liu et al. (2022); Cheng et al. (2024)

Omics Approaches	- Identification of thousands of ABA-responsive genes under drought, salinity, and heat stress using **high-throughput transcriptomics**.<br>- Discovery of ABA-regulated proteins and metabolites using **proteomics and metabolomics**.	**Transcriptomics**: RNA-seq for large-scale gene expression analysis.<br>**Proteomics**: Mass spectrometry for ABA-modulated proteins.<br>**Metabolomics**: Identification of ABA-regulated metabolites.	- Identification of key genes involved in ROS detoxification, osmotic regulation, and secondary metabolism.<br>- Unveiling of ABA-responsive pathways in diverse tissues and developmental stages.<br>- Discovery of potential biomarkers for stress tolerance.	- Integration of transcriptomics, proteomics, and metabolomics data remains fragmented.<br>- Need for **multi-omics integration** for a holistic view of ABA-regulated networks.<br>- Limited large-scale datasets for comparative omics across crop species.	Zhang et al. (2024); Liu et al. (2023)
Emerging Tools and Techniques	- Real-time visualization of ABA movement in cells using **live-cell imaging** and fluorescent sensors like **ABAleons**.<br>- Single-cell transcriptomics revealing cell-type-specific ABA responses.<br>- Machine learning for predictive modeling of ABA signaling networks.	**Live-Cell Imaging** for tracking ABA dynamics.<br>**ABAleons**: Fluorescent biosensors for real-time ABA quantification.<br>**Single-Cell Transcriptomics** to analyze gene expression in individual cells.<br>**Machine Learning (ML)** to predict ABA-responsive genes.	- Visualization of ABA concentration and movement at cellular resolution.<br>- Insights into tissue-specific ABA responses, especially in cell-type-heterogeneous tissues (e.g., root tips, epidermal cells).<br>- Use of ML to model ABA signaling networks and predict gene targets.	- Limited spatial resolution in single-cell analyses, especially for deep tissues.<br>- Dependence on large, high-quality datasets for effective **machine learning** predictions.<br>- Complexity in modeling ABA crosstalk with other hormone pathways.	Chen et al. (2023); Yu et al. (2023); Gong et al. (2023)
Integration of Advances for Crop Improvement	- New understanding of how ABA can be harnessed for improving crop drought tolerance.<br>- Use of ABA-responsive genes as **biomarkers** for crop selection and breeding.	**High-throughput Phenotyping** to screen ABA-responsive traits.<br>**Gene Editing (CRISPR/Cas9)** for crop improvement.	- Potential to breed crops with enhanced water-use efficiency and drought tolerance.<br>- Development of climate-resilient	- Need for crop-specific validation of ABA-responsive genes.<br>- Translating ABA research from model plants (e.g.,	Vaidya et al. (2022); Lu et al. (2024); Zhang et al. (2023)

		**Multi-omics Platforms** for holistic stress-tolerance profiling.	crops through gene editing and marker-assisted selection.	*Arabidopsis*) to polyploid crop species.<br>- **Ethical and regulatory challenges** in the deployment of gene-edited crops.	
